# A Phase I First-Time-in-Human, Open-label Study of Nelistotug Administered to Patients with Advanced Solid Tumors

**DOI:** 10.1158/1078-0432.CCR-25-4374

**Published:** 2026-08-27

**Authors:** Lillian L. Siu, Omid Hamid, Reva Schneider, Jordi Rodon Ahnert, Scott A. Laurie, Frank Stephen Hodi, Dong-Wan Kim, Myung-Ju Ahn, Gary Carlson, Hussein Assi, Hamza Khan, Patrick Hanafin, Emily Moore, Madhu Ramaswamy, Reza Mozaffari, Stefan Zajic, Arindam Dhar, Ivan Diaz Padilla, Noboru Yamamoto

**Affiliations:** 1 Princess Margaret Cancer Centre, Toronto, Canada.; 2 The Angeles Clinic and Research Institute Inc, A Cedars-Sinai Affiliate, Los Angeles, California.; 3 Mary Crowley Cancer Research Center, Sarah Cannon Research Institute at Mary Crowley, Dallas, Texas.; 4 The University of Texas MD Anderson Cancer Center, Houston, Texas.; 5 The Ottawa Hospital Cancer Centre, Ottawa, Canada.; 6 Dana-Farber Cancer Institute, Boston, Massachusetts.; 7 Department of Internal Medicine, Seoul National University College of Medicine, Seoul National University Hospital, Seoul, Republic of Korea.; 8 Samsung Medical Center, Sungkyunkwan University School of Medicine, Seoul, Republic of Korea.; 9 Oncology Clinical Development, GSK, Cambridge, Massachusetts.; 10 Oncology Clinical Development, GSK, Waltham, Massachusetts.; 11 Oncology Clinical Development, GSK, Mississauga, ON, Canada.; 12 Clinical Pharmacology, Modeling and Simulation, GSK, Collegeville, Pennsylvania.; 13 Development Oncology Biostatistics, GSK, Stevenage, United Kingdom.; 14 Oncology Translational Medicine, GSK, Rockville, Maryland.; 15 Biomarkers and Analytical Platforms, GSK, Collegeville, Pennsylvania.; 16 Oncology Clinical Development, GSK, Collegeville, Pennsylvania.; 17 Oncology Clinical Development, GSK, Baar, Switzerland.; 18 National Cancer Center Hospital, Tokyo, Japan.

## Abstract

**Purpose:**

This first-in-human, nonrandomized, multicenter study investigated nelistotug [anti–cluster of differentiation (CD) 96 monoclonal antibody (mAb)] as monotherapy (arm A), in combination with dostarlimab [anti–programmed cell death protein 1 (PD-1) mAb; arm B], and dostarlimab plus belrestotug [anti–T-cell immunoreceptor with Ig and immunoreceptor tyrosine-based inhibitory motif domains (TIGIT) mAb; arm F] in patients with recurrent or advanced solid tumors.

**Patients and Methods:**

Patients received intravenous doses every 3 weeks of nelistotug 2.1 to 1,400 mg, nelistotug 70 to 1,400 mg plus dostarlimab 500 mg, or nelistotug 1,400 mg plus dostarlimab 500 mg plus belrestotug 400 mg in arms A, B, and F, respectively. Primary endpoints were dose-limiting toxicities (DLT) and adverse events (AE). Secondary endpoints included objective response rate, pharmacokinetics, and immunogenicity. Disease control rate and CD96 receptor occupancy were exploratory.

**Results:**

Thirty-six, 33, and 3 patients were included in arms A, B, and F, respectively. No DLTs were observed in any arm, allowing for dose escalation to nelistotug maximum planned dose in arms A and B, with a recommended phase II dose of 1,400 mg. No treatment-related fatal AEs were observed. In arm B, 1 (3%) patient achieved a complete response and 1 (3%) achieved a partial response; no responses were observed in arms A and F. Nelistotug serum concentrations across doses were calculated to be sufficient to saturate the target in the tumor since >95% CD96 receptor occupancy was observed in peripheral blood immune cells.

**Conclusions:**

Nelistotug as monotherapy and in combination with dostarlimab and belrestotug had an acceptable safety and tolerability profile; however, efficacy was limited in this heavily pretreated patient population with advanced solid tumors.

**Translational Relevance**

Cluster of differentiation (CD)96 represents a novel target for cancer immunotherapy. In preclinical tumor models, CD96 inhibition demonstrated antitumor activity as monotherapy with enhanced activity when combined with other checkpoint inhibitors including, anti–programmed cell death protein 1 (PD-1) and anti–T-cell immunoreceptor with Ig and immunoreceptor tyrosine-based inhibitory motif domains (TIGIT) antibodies. Nelistotug is a monoclonal antibody (mAb) that inhibits CD96. This first-in-human dose-escalation study investigated nelistotug as monotherapy, in combination with dostarlimab (an anti–PD-1 mAb), and in combination with dostarlimab and belrestotug (an investigational anti-TIGIT antibody) in patients with recurrent or advanced solid tumors. The study met its primary endpoint with nelistotug as monotherapy and in combination, demonstrating an acceptable safety and tolerability profile. Antitumor activity was limited, with only 2 (6%) patients achieving a response, both in the nelistotug plus dostarlimab cohort. Near-complete CD96 receptor occupancy in peripheral blood immune cells was observed with nelistotug. Additional studies are required to understand CD96 as a potential therapeutic target, including specific tumor types and other/alternative combinations.

## Introduction

Immune checkpoint inhibitors (ICI) targeting the programmed cell death protein 1/ligand 1 [PD-(L)1] axis have shown clinical efficacy across multiple tumor types, but not all patients benefit from these therapies (1, 2). Tumors can overexpress redundant immunosuppressive pathways, leading to ICI resistance (1, 3, 4). There is a significant need to identify novel immune checkpoint therapeutic targets (2, 5).

**Figure 1.**
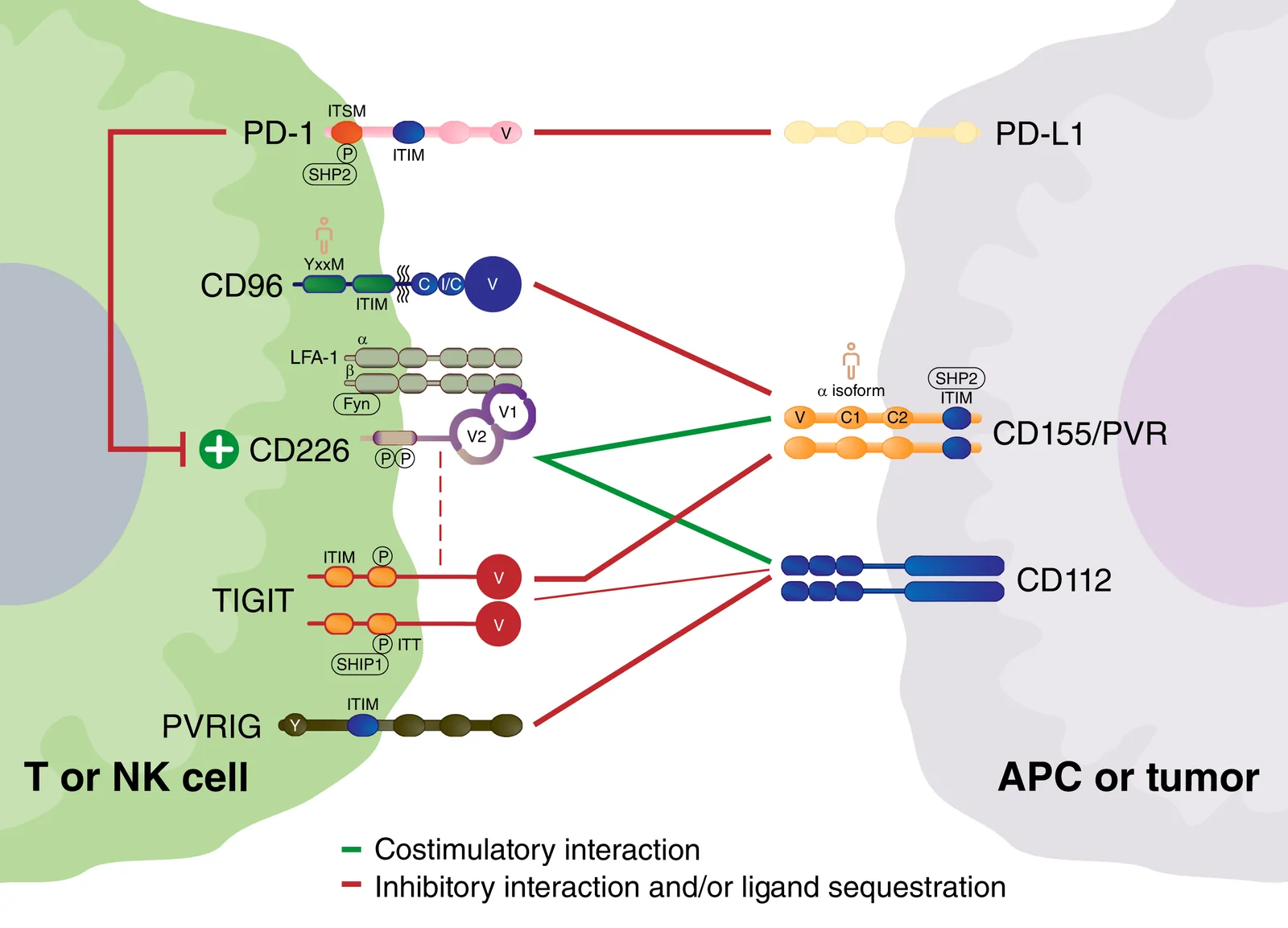
TIGIT/CD226 pathway. Dashed red lines indicate the potential for *cis* interactions/inhibition. The weight of each line is representative of the relative strength of interaction. Human silhouettes signify that a motif or isoform is not present in rodents. APC, antigen-presenting cell; C, constant-like domain; I/C, I/C-like folding pattern present in isoform 2 of human CD96; ITIM, immunoreceptor tyrosine-based inhibition motif; ITSM, immunoreceptor tyrosine-based switch motif; ITT, immunoglobulin tyrosine tail; LFA-1, lymphocyte function–associated antigen 1; P, tyrosine phosphorylation site; PVR, poliovirus receptor; PVRIG, poliovirus receptor–related immunoglobulin domain–containing protein; SHIP1, Src homology 2–containing inositol phosphatase 1; SHP2, Src homology 2–containing phosphotyrosine phosphatase; V, variable-like domain; Y, tyrosine residue. (Reproduced from Connor M, Hance KW, Yadavilli S, Smothers J, Waight JD. Emergence of the CD226 axis in cancer immunotherapy. Front Immunol 2022;13:914406, licensed under CC BY 4.0).

The cluster of differentiation (CD)226 axis plays an important role in natural killer (NK)–and T-cell biology and cancer immunosurveillance (6–8). The CD226 immune-activating receptor is expressed on NK and T cells, and binds to the CD155 and CD112 ligands expressed on tumor and antigen-presenting cells, stimulating an immune response (6, 7). Conversely, CD96 and T-cell immunoreceptor with Ig and immunoreceptor tyrosine-based inhibitory motif domains (TIGIT) compete with CD226 for binding to CD155 and CD112 to suppress immune activation (Fig. 1; refs. 7, 8). CD96 has been shown to regulate NK-cell effector function and tumor metastasis and represents a novel target for cancer immunotherapy (9). In tumor models, targeting CD96 resulted in antitumor activity and was shown to be more effective when other ICIs, including PD-1, PD-L1, TIGIT, and cytotoxic T lymphocyte–associated protein 4 (CTLA-4), were also targeted (9–11). An association of CD96 expression with other immune checkpoints, including coexpression of CD96 with PD-1 on CD8^+^ tumor-infiltrating lymphocytes, has been reported (9, 12). Additionally, preclinical data demonstrate superior immune activation and antitumor activity with a human Fc-enhanced bispecific antibody against the TIGIT and CD96 coreceptor, compared with anti-TIGIT or anti-CD96 monoclonal antibodies (mAb; ref. 13).

Nelistotug (GSK6097608) is a mAb that has been shown to block CD96 function, enhancing CD155/CD226 activation of NK/T cells (14, 15). A first-in-human, phase I, dose-escalation study was conducted to assess nelistotug as monotherapy and in checkpoint inhibitor combinations in patients with recurrent or advanced solid tumors. Results presented here are from study arms that evaluated nelistotug as monotherapy; in combination with dostarlimab, an anti–PD-1 mAb approved in advanced/recurrent endometrial cancer and in advanced/recurrent mismatch repair–deficient tumors (16); and in combination with dostarlimab plus belrestotug, an investigational anti-TIGIT mAb (17, 18).

## Patients and Methods

### Study design

This study (ClinicalTrials.gov identifier: NCT04446351) was a first-in-human, open-label, nonrandomized, multicenter study to investigate the safety, tolerability, pharmacokinetics (PK), pharmacodynamics (PD), immunogenicity, and preliminary anticancer activity of nelistotug monotherapy administered intravenously in patients with recurrent or advanced solid tumors and in combination with other checkpoint inhibitors. The study included a dose-escalation portion, driven by clinical safety and supplemented by PK and PD [receptor occupancy (RO)] data to determine the recommended phase II dose (RP2D) of nelistotug as monotherapy (arm A) and in combination with dostarlimab (arm B; Supplementary Fig. S1). The RP2D criteria for CD96 were a dose-limiting toxicity (DLT) rate <33% and RO ≥80% on CD3+ peripheral blood mononuclear cells (PBMC) at C_trough_ in ≥75% of evaluable patients. An additional PK/PD portion evaluated nelistotug at the RP2D in specific tumor types. Dose expansion was not performed. The study was designed with additional treatment arms to support the potential for future studies in specific Asian populations; dostarlimab monotherapy (arm D), dostarlimab in combination with belrestotug (arm E), dostarlimab, belrestotug, and nelistotug combination therapy (arm F), and dostarlimab in combination with cobolimab (arm G) were evaluated in Chinese or Japanese patients. Only results from nelistotug-containing arms are presented here: arms A and B (dose escalation of nelistotug as monotherapy and in combination with dostarlimab, respectively) and arm F (exploratory; supporting future clinical studies in Japan; no dose escalation planned). This was a phase I open-label study with no blinding or randomization.

The study was conducted in accordance with the Declaration of Helsinki and Good Clinical Practice guidelines following approval by ethics committees and institutional review boards at each study site. All patients provided written informed consent.

### Study population

Eligible patients were ≥18 years of age (or ≥20 years for patients enrolled in Japan) at the time of consent, with histologic or cytologic documentation of locally advanced, recurrent, or metastatic solid malignancy. The disease must have progressed after standard therapy for the specific tumor type, or standard therapy was ineffective, intolerable, or considered inappropriate, or no further standard therapy existed. Prior cancer therapy could have included chemotherapy, immunotherapy (except those targeting CD96), biological therapy, targeted therapy, hormonal therapy, or investigational agents. The arm A PK/PD cohort was restricted to non–small cell lung cancer (NSCLC) and head and neck squamous cell carcinoma (HNSCC), whereas the arm B PK/PD cohort was restricted to endometrial cancer. Eligible patients also required an Eastern Cooperative Oncology Group performance status 0–1 and adequate organ function.

Patients were excluded if they had received prior treatment with the following therapies: any therapy directed against CD96; ICIs within 4 weeks (from the last dose of prior treatment to the first dose of study intervention); and other anticancer therapy (including chemotherapy, targeted therapy, and biological therapy) within 4 weeks.

### Treatment and dosing

Nelistotug, dostarlimab, and belrestotug were administered intravenously at fixed doses. For the dose-escalation phase, a 3 + 3 dose-escalation procedure guided dose-escalation decisions informed by the Neuenschwander continual reassessment method (N-CRM; except in Japan, where decisions were based on the 3 + 3 guidelines only). N-CRM data and the 3 + 3 data from Japanese patients were evaluated separately and in a pooled analysis; the pooled analysis is presented here. Dose escalation was based primarily on DLTs, although clinical safety assessments, PK, and PD data were also considered. N-CRM informed dose-escalation decisions using Fixed and Adaptive Clinical Trial Simulator (FACTS) software (version 6.1). The N-CRM design uses a Bayesian logistic regression model relating dose and toxicity to locate the MTD efficiently while minimizing the number of patients exposed to pharmacologically inactive or unsafe dose levels; it is a modified version of the continuous reassessment method (19, 20). The N-CRM supported dose escalation or de-escalation depending on posterior DLT probability ranges: under-dosing (0%–16%), target toxicity (16%–33%), excessive toxicity (33%–60%), or unacceptable toxicity (>60%).

The starting dose of nelistotug for dose escalation was determined using a minimally anticipated biological effect level approach to identify a dose with 10% to 30% of predicted maximal pharmacologic activity in a plate-bound CD155 assay measuring nelistotug-mediated rescue of interferon (IFN) γ production by human PBMCs. Other pharmacologically relevant experiments to support the starting dose included cell-based *in vitro* binding, ligand binding inhibition, and ligand (CD155) binding disruption (representing RO). A starting dose of 2.1 mg every 3 weeks was estimated to provide a plasma C_max_ at steady state (determined from simulations using human PK parameters derived by allometrically scaling PK parameters from a dose range–finding study in cynomolgus monkeys) that corresponded to 25.5% of the maximal IFNγ production in the plate-bound CD155 PBMC assay, 80% ligand binding disruption, 97.6% ligand binding inhibition, and a predicted RO level of 99.2%.

Dose-escalation levels for nelistotug monotherapy in arm A were 2.1, 7, 21, 70, 210, 700, and 1,400 mg every 3 weeks, with each dose level increase based on a maximum half-log (i.e., 3.2-fold) increase. The dose-escalation phase evaluating the combination of nelistotug and dostarlimab in arm B was initiated after a tolerable nelistotug monotherapy dose with adequate exposure to ensure pharmacologic activity based on PK data was identified. The starting dose of nelistotug in combination with dostarlimab was at least 1 dose level below the identified nelistotug monotherapy dose; the doses for nelistotug were 70, 210, 700, and 1,400 mg every 3 weeks. Dostarlimab was administered at a fixed dose of 500 mg every 3 weeks. Arm F was initiated after a review of data from other treatment arms of the current study and from preliminary clinical data of separate studies, including ENTRÉE Lung (GSK 205801; ClinicalTrials.gov identifier: NCT03739710; ref. 21) and GALAXIES Lung-201 (GSK 213824; ClinicalTrials.gov identifier: NCT05565378; ref. 18). There was no dose escalation for arm F; this was performed in the ENTRÉE Lung study. Dosing was nelistotug 1,400 mg every 3 weeks, dostarlimab 500 mg every 3 weeks, and belrestotug 400 mg every 3 weeks. A PK/PD cohort was initially planned for arm F if responses were observed in the arm E (dostarlimab + belrestotug) PK/PD cohort; however, no responses were observed. Study treatments were administered for up to 2 years or until disease progression, unacceptable toxicity, death, or withdrawal of consent.

### Endpoints

The primary endpoints were incidence of DLTs and incidence, duration, and severity of adverse events (AE) and serious AEs (SAE) to characterize the safety profile and tolerability of nelistotug as a monotherapy (arm A), in combination with dostarlimab (arm B), and in combination with dostarlimab and belrestotug (arm F). These also informed the RP2D of nelistotug as monotherapy and in combination with dostarlimab.

Secondary endpoints included further characterization of safety and tolerability, including incidence of dose modifications (e.g., dose reductions or delays) and discontinuations due to AEs; objective response rate (ORR) based on Response Evaluation Criteria in Solid Tumors, version 1.1 (RECIST 1.1) criteria; PK for nelistotug; and immunogenicity [incidence and titers of antidrug antibody (ADA) to nelistotug, dostarlimab, or belrestotug as indicated by treatment arm].

Exploratory endpoints included disease control rate (DCR) defined as the percentage of patients with a confirmed complete response (CR) or partial response (PR) at any time, or stable disease (SD) ≥12 weeks based on RECIST 1.1, and CD96 RO by direct binding of nelistotug to CD96.

### Assessments

In the dose-escalation portion, DLTs (defined in Supplementary Methods) were assessed during cycle 1 (21 days), the DLT observation period. Safety assessments included physical examinations, vital signs, electrocardiograms, echocardiograms, laboratory assessments, oxygen saturation, and chest imaging for the assessment of interstitial lung disease (arm F only). AEs were coded using the standard Medical Dictionary for Regulatory Activities and graded by the investigator according to the National Cancer Institute Common Terminology Criteria for Adverse Events (version 5.0). AEs were collected during study treatment, with SAEs and immune-related (ir) AEs (AEs of special interest) collected until 30 or 90 days after the last dose of study treatment depending on the initiation of a subsequent therapy.

Tumor response was assessed according to RECIST 1.1 by the investigator. Disease assessments included imaging of the chest, abdomen, and pelvis by intravenous contrast-enhanced computed tomography (CT; preferred) or non–contrast-enhanced CT scans of the chest plus contrast-enhanced magnetic resonance imaging of the abdomen and pelvis for patients sensitive to CT contrast. Assessments were performed at baseline and then every 6 weeks up to week 24, every 9 weeks from week 33 to week 51, and every 12 weeks thereafter, or at the time of treatment discontinuation unless one had been conducted within 6 weeks.

Blood and serum samples were collected at baseline and scheduled timepoints to evaluate the PK of study drugs, as well as ADAs and biomarkers. A custom flow assay was used to assess CD96 RO in whole blood. The panel included T- and NK-cell lineage markers in addition to a noncompetitive anti-CD96 antibody (GSK CD96 14D3 clone conjugated to phycoerythrin) to measure total CD96 and a competitive anti-CD96 antibody (GSK6097608B_PE) to measure free CD96. The ratio of total to free CD96 was used to calculate CD96 RO. For tumor PD assessment, RNA sequencing was performed on screening/pretreatment and week 6 posttreatment biopsy samples from the arm B PK/PD cohort (Supplementary Methods).

### Statistical analysis

The safety population included all patients who received ≥1 dose of study intervention. Unless otherwise specified, the safety population was used for all study population, efficacy, and safety analyses. The DLT-evaluable population included all patients who received ≥1 dose of study intervention and were followed for the DLT observation period. Patients enrolled in a PK/PD cohort at a previously cleared dose were not included in the DLT-evaluable population. PK, PD, and immunogenicity were analyzed for all evaluable samples. Statistical differences and trends in immune modulation at different doses were determined by calculating the average logarithmic 2-fold change normalized to baseline (day 1 predose).

There was no formal hypothesis testing. Unless otherwise specified, continuous and categorical data were summarized using descriptive statistics. AEs were summarized by frequency and proportion of total patients, system organ class, and preferred term.

The planned sample sizes for the dose-escalation and PK/PD portions were approximately 72 and 52 patients in arms A and B, respectively, and up to 15 DLT-evaluable patients in arm F.

## Results

### Study patients

**Figure 2.**
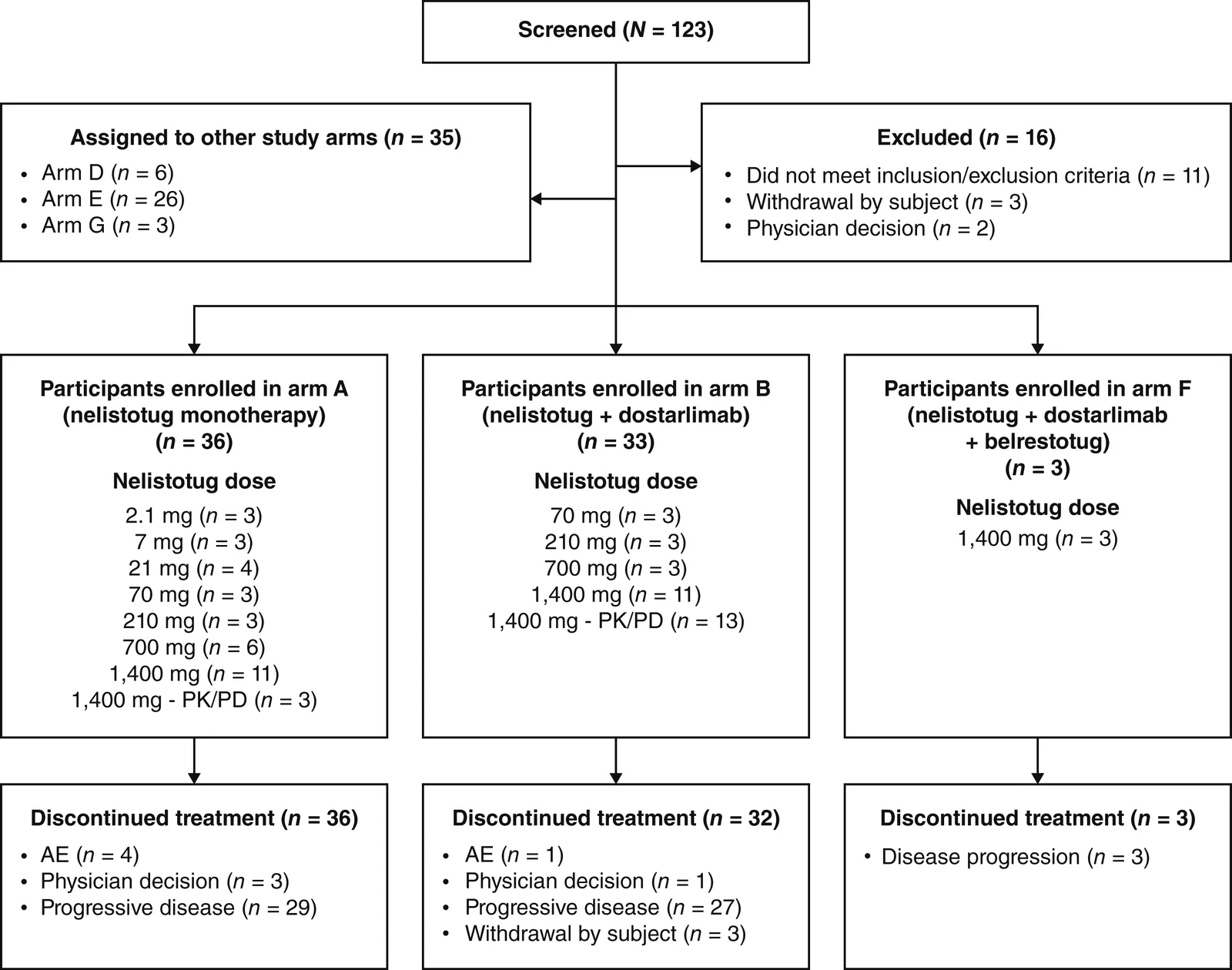
Patient disposition.

The study was conducted at 11 centers in 4 countries (Canada, Japan, the Republic of Korea, and the United States). As of data cutoff (February 4, 2025), 72 patients were enrolled and had received at least 1 dose of a study drug across arms A, B, and F (36, 33, and 3 patients, respectively; Fig. 2). All three patients in arm F were enrolled in Japan for the evaluation of DLT and safety; as no DLTs were observed in these patients and no additional dose escalation was planned, no additional patients were enrolled in arm F.

In arms A and B, the majority of patients were White (72% and 67%, respectively), followed by Asian race (22% and 24%, respectively; Table 1). The most common primary tumor types were head and neck cancer (25%), colon cancer (14%), and pancreatic carcinoma (11%) in arm A and endometrial cancer (42%), head and neck cancer (15%), and colon cancer (12%) in arm B. Full listings of primary tumor types are provided in Supplementary Table S1. The median time since initial diagnosis was 3 years in both arms. Most patients had metastatic disease at baseline (83% in arm A and 85% in arm B) and had received ≥3 regimens of prior anticancer therapies (78% and 63%, respectively). In addition, 44% and 58% of patients, respectively, had prior radiotherapy, and prior immunotherapy had been received by 61% and 64%, respectively (Table 1). Baseline characteristics for arm F are shown in Supplementary Table S2. Primary tumors were colon cancer, NSCLC, and uterine cancer. The representativeness of study patients is shown in Supplementary Table S3. At the time of the data cutoff, 71 of 72 patients had discontinued treatment, primarily due to progressive disease (59 patients; Fig. 2). One patient in arm B completed 2 years of treatment.

**Table 1.** Baseline patient and disease characteristics.

Baseline characteristic	Nelistotug monotherapy (arm A), *N* = 36	Nelistotug + dostarlimab (arm B), *N* = 33
Age, years^a^	​	​
Median (range)	63.5 (37–85)	62 (28–79)
Sex, *n* (%)	​	​
Male	19 (53)	8 (24)
Female	17 (47)	25 (76)
High-level race, *n* (%)	​	​
White	26 (72)	22 (67)
Asian	8 (22)	8 (24)
Black or African American	2 (6)	3 (9)
Ethnicity, *n* (%)	​	​
Hispanic or Latino	6 (17)	5 (15)
Not Hispanic or Latino	30 (83)	28 (85)
Time since initial diagnosis (years)^b^	​	​
Median (range)	3 (1–27)	3 (0–14)
Stage at screening, *n*/*N* (%)	​	​
Ib	0	1/32 (3)
IIa	0	1/32 (3)
III^c^	3/33 (8)^d^	2/32 (6)^d^
IV^c^	30/33 (83)^d^	28/32 (85)^d^
Primary tumor type, *n* (%)^e^	​	​
Head and neck	9 (25)	5 (15)
Colon	5 (14)	4 (12)
Pancreatic	4 (11)	—
Neoplasms of the parotid gland	4 (11)	—
Endometrial	—	14 (42)
NSCLC	—	—
Uterine	—	—
Number of prior anticancer therapy regimens, *n* (%)	​	​
1	3 (8)	5 (15)
2	5 (14)	7 (21)
3	9 (25)	8 (24)
4	8 (22)	2 (6)
>4	11 (31)	11 (33)
Median (range)	4 (1 to >4)	3 (1 to >4)
Prior immunotherapy, *n* (%)	22 (61)	21 (64)

a Age is imputed when full date of birth not provided.

b Time since initial diagnosis (years) is defined as screening visit year minus initial diagnosis year.

c Includes stages IIIa and IIIc under III and stages IVa, IVb, and IVc under IV.

d Percent values calculated based on total denominator (*N* = 36 for arm A and *N* = 33 for arm B).

e Only primary tumor types in >10% patients are shown.

### Treatment exposure

A summary of exposure by dose level, including dostarlimab and belrestotug, is shown in Supplementary Table S4. The median dose of nelistotug in arm A was 690 mg (range, 1.9–1,423) every 3 weeks, with a median of 2.5 (range, 1–25) infusion cycles and a median duration of exposure of 7.5 (range, 3–75) weeks. In arm B, the median dose intensity for nelistotug was 1,384 mg (range, 70–1,434) every 3 weeks, with a median of 3 (range, 1–34) infusion cycles and a median duration of exposure of 9 (range, 3–108) weeks.

### Safety

The DLT-evaluable population comprised 33 patients in arm A, 19 patients in arm B, and 3 patients in arm F, with no DLTs in any arm and no apparent dose-dependent trends in AEs. Based on clinical safety, PK, and PD data, the RP2D for nelistotug was 1,400 mg for both monotherapy (arm A) and the combination with dostarlimab (arm B).

In the safety populations of arms A and B, 33 (92%) and 29 (88%) patients, respectively, experienced treatment-emergent AEs (TEAE; Table 2). In arm F, all 3 patients experienced a TEAE. There were no treatment-related SAEs or fatal AEs in any of the arms.

**Table 2.** Summary of AEs.

Adverse events	Nelistotug monotherapy (arm A), *N* = 36	Nelistotug + dostarlimab (arm B), *N* = 33
Any AE, *n* (%)^a^	33 (92)	29 (88)
AEs related to study treatment	20 (56)	20 (61)
Grade ≥3 AEs	14 (39)	10 (30)
Grade ≥3 AEs related to study treatment	1 (3)	0
AEs leading to permanent discontinuation of study treatment	4 (11)	2 (6)
TRAEs leading to permanent discontinuation of study treatment	1 (3)	1 (3)
AEs leading to dose interruption/delay	4 (11)	5 (15)
Any SAE, *n* (%)^a^	7 (19)	7 (21)
Treatment-related SAEs	0	0
Grade ≥3 SAEs	7 (19)	7 (21)
Fatal SAEs	0	0
Treatment-related fatal SAEs	0	0

a Discontinuation of treatment reflects discontinuation of at least one component of study treatment administered; relationship to treatment reflects relationship to at least one component.

Four (11%) patients in arm A and 5 (15%) patients in arm B had a dose interruption or delay to manage TEAEs. Only 1 (3%) patient in arm A (decreased appetite) and 1 (3%) in arm B (hypertransaminasemia) discontinued study treatment because of a treatment-related AE (TRAE). In arm F, no patient experienced a TEAE leading to dose modification or study treatment discontinuation.

Table 3 summarizes TEAEs occurring in >10% of patients overall in any treatment arm. Grade ≥3 TEAEs occurred in 14 (39%) patients in arm A and included grade 4 hypovolemic shock in 1 patient and grade 4 pulmonary hemorrhage in 1 patient. In arm B, 10 (30%) patients experienced a grade ≥3 TEAE, including grade 4 hyponatremia, meningitis, sepsis, thalamic infarction, and tumor hemorrhage. TEAEs for arm F are shown in Supplementary Table S5. No Grade ≥3 TEAEs were reported in arm F.

**Table 3.** TEAEs of any grade occurring in >10% of patients^a^.

Preferred term, *n* (%)	Nelistotug monotherapy (arm A), *N* = 36	Nelistotug + dostarlimab (arm B), *N* = 33	Total (*N* = 72)^b^
Any event	33 (92)	29 (88)	65 (90)
Fatigue	12 (33)	8 (24)	20 (28)
Constipation	5 (14)	9 (27)	15 (21)
Headache	2 (6)	11 (33)	13 (18)
Nausea	9 (25)	3 (9)	13 (18)
Decreased appetite	7 (19)	4 (12)	12 (17)
Diarrhea	6 (17)	6 (18)	12 (17)
Anemia	5 (14)	4 (12)	9 (13)
Pruritus	2 (6)	6 (18)	9 (13)
Rash	3 (8)	5 (15)	9 (13)
Chills	4 (11)	3 (9)	8 (11)
Cough	2 (6)	6 (18)	8 (11)
Pyrexia	1 (3)	7 (21)	8 (11)
Abdominal pain	2 (6)	5 (15)	7 (10)
Arthralgia	3 (8)	4 (12)	7 (10)
Dyspnea	3 (8)	4 (12)	7 (10)
Vomiting	4 (11)	2 (6)	6 (8)
Dizziness	4 (11)	1 (3)	5 (7)
Hypokalemia	4 (11)	1 (3)	5 (7)
Insomnia	4 (11)	0	5 (7)
Infusion-related reaction	0	4 (12)	5 (7)
Lipase increased	1 (3)	1 (3)	4 (6)

a TEAEs occurring in >10% patients in any treatment arm.

b Includes 3 patients from arm F.

TRAEs were observed in 20 (56%) patients in arm A, 20 (61%) patients in arm B, and 3 (100%) patients in arm F. Only 1 patient experienced a TRAE of grade ≥3 (fatigue), which was reported in arm A (Table 2). The most common TRAEs observed in >10% patients were fatigue (*n* = 7, 19%; *n* = 6 grade 1–2; *n* = 1 grade 3) and grade 1 chills (*n* = 4, 11%) in arm A and grade 1 to 2 headache (*n* = 7, 21%), grade 1 to 2 fatigue (*n* = 5, 15%), grade 1 to 2 rash (*n* = 5, 15%), grade 1 to 2 infusion-related reaction (*n* = 4, 12%), and grade 1 pruritus (*n* = 4, 12%) in Arm B. Grade 1 to 2 infusion-related reaction occurred in 4 (12%) patients in arm B and 1 (33%) patient in arm F. Time to onset of the first occurrence from day of infusion was ≤1 day in both arm B and arm F. No infusion-related reactions were observed in arm A.

irAEs developed in 1 patient in arm A (grade 2, adrenal insufficiency, alanine aminotransferase increased, and aspartate aminotransferase increased). In arm B, 4 patients experienced irAEs (all grade 2), including rash in 2 patients, hypothyroidism in 1 patient, and arthralgia in 1 patient. In arm F, 1 patient experienced an irAE (grade 2 rash). None of the irAEs were assessed as serious, and none led to discontinuation of study treatment. No novel irAEs were reported.

### Antitumor activity

In arms A and F, there were no confirmed responses. In the nelistotug 210-mg group of arm B, 1 patient with esophageal squamous cell carcinoma had a PR and 1 patient with HNSCC had a CR, for an ORR of 6.1% with a duration of response of 105 and 58 weeks, respectively. A substantial proportion of patients in arms A (50%) and B (27%) experienced SD as best response. SD was reported across multiple tumor types, most commonly HNSCC in arm A (*n* = 8, 44%) and endometrial cancer in arm B (*n* = 4, 44%). DCRs were 27.8% and 30.3%, for arms A and B, respectively (Table 4). Supplementary Table S6 summarizes responses by nelistotug dose level for arms A and B, and arm F.

**Table 4.** Tumor response (RECIST 1.1).

Confirmed responses	Nelistotug monotherapy (arm A), *N* = 36	Nelistotug + dostarlimab (arm B), *N* = 33
Best overall response, *n* (%)	​	​
CR	0	1 (3)
PR	0	1 (3)
SD	18 (50)	9 (27)
SD ≥12 weeks	10 (28)	8 (24)
PD	16 (44)	20 (61)
Nonevaluable^a^	2 (6)	2 (6)
ORR	​	​
CR + PR, % (95% CI)^b^	0 (0–9.7)	6.1 (0.7–20.2)
DCR^c^	​	​
CR + PR + SD (≥12 weeks), % (95% CI)^b^	27.8 (14.2–45.2)	30.3 (15.6–48.7)

Abbreviation: CI, confidence interval; PD, progressive disease.

a Patients who had at least one post-baseline response assessment and none of the investigator-assigned responses qualified to be CR, PR, SD, or PD based on the defined criteria.

b Two-sided 95% exact (Clopper–Pearson) CIs are provided.

c DCR is defined as the percentage of patients with a confirmed CR or PR at any time, or SD ≥12 weeks.

### Pharmacokinetics (nelistotug)

All 72 patients had evaluable samples and were included in PK analyses. Serum concentration–time profiles of nelistotug in arms A and B are shown in Supplementary Fig. S2. Across the dose range (2.1–1,400 mg), there was a trend toward a greater than dose-proportional increase in nelistotug monotherapy exposure at lower doses and a close-to-proportional increase at doses >70 mg; nelistotug doses ≥210 mg were in the linear range. Nelistotug PK exhibited generally moderate variability [<60% coefficient of variation (CV) for AUC and C_max_ parameters] and did not seem to be affected in the combinations. For arms A and B (Supplementary Tables S7 and S8), the geometric mean (GM) exposure of nelistotug increased with increasing dose. GM exposures were similar when nelistotug was administered alone in arm A and in combination with dostarlimab 500 mg in arm B. However, at nelistotug 70 mg in combination with dostarlimab, GM exposure tended to be slightly higher, but this was accompanied by higher interpatient variability in arm B compared with arm A (% CV, 42%–60% and 38%–40%, respectively). When nelistotug was administered with dostarlimab 500 mg and belrestotug 400 mg in arm F, the GM exposure was higher compared with nelistotug alone in arm A or in combination with dostarlimab in arm B (Supplementary Table S9). The GM accumulation of nelistotug was minimal to moderate based on a range of 1.2 to 2.8 for the area under the curve ratio and a range of 0.8 to 5.3 for the C_max_ ratio (RC_max_) across treatment arms and doses. The median t_max_ values were generally similar across nelistotug doses for arms A and F but seemed to occur slightly later in arm B. The GM half-life seemed to be shorter at the lower doses of nelistotug but was generally similar across treatment arms. In general, the clearance (CL) of nelistotug monotherapy in arm A was similar across most doses but seemed higher at the lowest dose of 2.1 mg. Coadministration with dostarlimab in arm B did not seem to affect nelistotug CL, whereas nelistotug CL seemed lower when coadministered with dostarlimab and belrestotug in arm F.

### Immunogenicity

Immunogenicity was assessed using an appropriate risk-based bioanalytic assay strategy consisting of screening, confirmation, and titration assays to detect anti-nelistotug, anti-dostarlimab, and anti-belrestotug antibody responses. In arm A, all 36 patients had at least 1 immunogenicity sample collected with an analysis result. Overall, 2 of 36 (6%) patients had treatment-emergent anti-nelistotug antibodies, with a median titer value of 80 (range, 40–320). In arm B, 32 patients were tested for anti-nelistotug antibodies and 31 patients were tested for anti-dostarlimab antibodies. All 32 (100%) patients were negative at baseline and after baseline for anti-nelistotug antibodies. Two (6%) patients had treatment-emergent anti-dostarlimab antibodies.

### Biomarkers

Individual patient-level data for CD96 RO frequency within CD4+ and CD8+ T cells and CD56+ NK cells are shown in Supplementary Fig. S3, across multiple timepoints for all tested doses of nelistotug in arms A and B. In arm A, RO (>95%) was observed across all dose cohorts, starting from a 21-mg dose of nelistotug, and was maintained across the dosing interval. At doses <21 mg, full CD96 RO was not maintained during the 21-day dosing interval (Supplementary Fig. S3A). CD96 RO (>95%) was also observed in all arm B–tested patients of the dose-escalation cohort (Supplementary Fig. S3B) and arm B PK/PD patients (Supplementary Fig. S3C) across all timepoints. The observed CD96 RO on peripheral NK- and T-cell populations indicates sustained target engagement with nelistotug doses ≥21 mg every 3 weeks administered as monotherapy and in combination with dostarlimab.

Individual total soluble CD96 data for arm A are presented in Supplementary Fig. S4. Total soluble CD96 levels were observed to increase within the first 4 hours after treatment, indicating binding of nelistotug to the CD96 target and increased levels of the mAb–target complex. At nelistotug doses ≤70 mg every 3 weeks, total soluble CD96 levels began to decrease by week 3, which indicated lower levels of the mAb–target complex and hence lower levels of target engagement compared with higher doses. This is reflective of lower nelistotug trough concentrations at doses ≤70 mg every 3 weeks. At nelistotug doses of ≥70 mg every 3 weeks, total soluble CD96 levels were observed to peak or saturate at week 3 and later. This is reflective of continued target engagement by nelistotug throughout the dose interval at higher dose levels and drug concentrations.

Peripheral PD activity was not observed in the monotherapy or combination arms. Tumor PD assessment was done on matched baseline and week 6 biopsy samples only from 8 patients in arm B PK/PD. Data from tumor biopsies indicated that nelistotug in combination with dostarlimab significantly activated immune components within the tumor microenvironment. Multiple immune-related pathways and T-cell signatures were upregulated, including enrichment for CD8^+^ and CD4^+^ T-cell activation signatures, indicative of immune activation within the tumor microenvironment. The CD226 axis genes (*CD96*, *TIGIT*, *PVR*, and *Nectin 2*) and some associated genes (*PD-1* and *PD-L1*) were not upregulated in posttreatment tumor samples (Supplementary Fig. S5).

## Discussion

This first-time-in-human, open-label, nonrandomized study investigated the initial safety, tolerability, and preliminary anticancer activity of nelistotug, as monotherapy (arm A), in combination with dostarlimab (arm B), and in combination with dostarlimab plus belrestotug (arm F), and was the first clinical study to evaluate the inhibition of the CD96 pathway with immunotherapy. This was a heavily pretreated patient population across a variety of advanced tumors to enable the identification of potential activity signals in specific tumor types. Most patients had received multiple lines of prior anticancer therapy, and most had received prior immunotherapy.

Nelistotug as monotherapy, in combination with dostarlimab, and in combination with dostarlimab and belrestotug, demonstrated an acceptable safety and tolerability profile. There were no DLTs in the dose-escalation portion in arms A or B and no apparent dose-dependent trends in AEs, which allowed for dose escalation of nelistotug to the maximum planned dose in both arms. PK data indicated that nelistotug doses ≥210 mg were in the linear range, and peripheral RO ≥ 80% at C_trough_ was achieved in ≥75% of evaluable patients at doses ≥21 mg. Additionally, the rate and severity of clinical safety events indicated that the highest tested dose of 1,400 mg would be selected as the RP2D for nelistotug as monotherapy and in combination with dostarlimab. Despite the RO assay indicating effective peripheral target engagement, tumor target engagement could not be established because of the lack of tumor PD effects from the monotherapy arm. No DLTs were reported for nelistotug plus dostarlimab plus belrestotug in arm F. The nelistotug safety profile in the monotherapy arm was generally consistent with that of other checkpoint inhibitors targeting the CD226 axis, and the safety profiles of the combinations were consistent with known toxicities of therapies targeting PD-(L)1 or TIGIT. No novel irAEs were observed in any of the three treatment arms (22–24).

Efficacy data showed modest antitumor activity, with few patients achieving a confirmed response. One patient experienced a PR and another a CR, both in arm B at a 210-mg dose of nelistotug. There were no responses in arms A and F. A substantial proportion of patients in arms A (50%) and B (27.3%) experienced SD as best response. Due to the limited efficacy observed in the dose-escalation cohorts, the tumor types in the PK/PD cohorts were restricted to NSCLC and HNSCC in arm A and endometrial cancer in arm B. The lack of stratified analyses by tumor type is a limitation of the study. Because the overall numbers in the PK/PD cohorts were small, it is difficult to derive meaningful conclusions. Furthermore, as limited responses were observed in arm B, only 3 patients were enrolled in this cohort to assess DLTs.

Nelistotug exhibited linear, dose-proportional PK at doses of ≥70 mg every 3 weeks with moderate variability. This study was not designed to explore differences in PK based on tumor type. Nelistotug PK did not vary significantly when nelistotug was coadministered with dostarlimab or dostarlimab plus belrestotug. Nelistotug concentrations following administration as a monotherapy or in combination with dostarlimab resulted in >95% CD96 RO on peripheral blood immune cells in most patients. Total soluble CD96 levels increased immediately after treatment, indicating binding of nelistotug to the CD96 target and increased levels of the mAb–target complex. The target seemed saturated across the dosing interval at nelistotug doses above 70 mg every 3 weeks. Taken together, these findings indicate efficient peripheral target engagement, which may be suggestive of CD96 engagement at the tumor site. The risk of immunogenicity was low, with no observed impact on safety, efficacy, or PK.

In biomarker analyses, nelistotug alone or in combination with dostarlimab did not show any peripheral PD activation. However, there was an enrichment of immune pathways and T-cell activation in tumor samples at week 6 of treatment versus baseline with nelistotug plus dostarlimab in arm B, whereas NK signatures or NK activation pathways (high expressers of CD96 in tumor) were unchanged. There was also no significant change in the expression of multiple CD226 axis genes in arm B at week 6 and no association with DCRs. However, the tumor PD data are derived from a small set of heavily pretreated patients (*n* = 8) with limited clinical response. Furthermore, only 3 patients were enrolled into the PK/PD cohort of nelistotug monotherapy (arm A), and post-baseline tumor biopsies were not collected. The PD dataset does not support confirmation of nelistotug’s biological activity.

Collectively, the data reported here suggest that nelistotug as monotherapy and in combination with dostarlimab has an acceptable safety and tolerability profile in patients with advanced or recurrent solid tumors. However, despite the strong preclinical rationale for targeting CD96 in the context of PD-(L)1 inhibition (11, 12), efficacy across treatment arms was limited, which may reflect the limitations of CD96 as a therapeutic target in a molecularly unselected patient population. This was a heavily pretreated study population, and prior treatment, which included anti–PD-(L)1 inhibitors, can shift the tumor microenvironment to a more immunosuppressive state resulting in resistance to subsequent ICI therapy (25). Prior response to anti–PD-(L)1 inhibitors may have informed our observations; however, this prior response information was unavailable and reflects a limitation of the study. Intratumor heterogeneity along with multiple lines of therapy can lead to T-cell exhaustion, compounded expression of immune checkpoints, and attenuation of the immune response (25–27). Within the TIGIT/CD96/CD226 axis, TIGIT is likely to be the dominant inhibitory receptor on both NK and T cells (5), and CD96 expression may differ from that of PD-1 and TIGIT. Therefore, targeting CD96 with nelistotug monotherapy may be insufficient to overcome TIGIT- or PD-(L)1–driven immunosuppression. Promising preclinical data demonstrated that dual targeting of CD96 and TIGIT, in combination with PD-1/PD-L1 inhibition, may enhance antitumor immune responses (9, 13, 28). However, several TIGIT-based therapies have struggled to demonstrate relevant efficacy in late-stage clinical trials (29, 30). The phase III SKYSCRAPER-01 study evaluating the anti-TIGIT antibody tiragolumab in combination with the PD-L1 inhibitor atezolizumab failed to improve the primary endpoints of progression-free survival and overall survival in patients with previously untreated PD-L1–high, locally advanced NSCLC (29). These collective findings underscore the current limitations of therapeutic strategies targeting the TIGIT pathway (31). In the present study, the response to the combination of nelistotug, dostarlimab, and belrestotug may have been attenuated by the expression of additional immune checkpoint pathways (e.g., LAG-3 or CTLA-4; ref. 4). Further studies are needed to understand the potential therapeutic role of nelistotug and other CD96-targeting approaches, as monotherapy or in combination with other immunotherapies, across cancer types, prior therapies, and biomarkers including immune checkpoint expression.

## Supplementary Material

Supplementary Material1 Supplementary Material1

## Data Availability

GSK makes available anonymized individual patient data and associated documents from interventional clinical studies which evaluate medicines upon approval of proposals submitted to https://www.gsk-studyregister.com/en/. To access data for other types of GSK-sponsored research, for study documents without patient-level data, and for clinical studies not listed, please submit an enquiry via the website.
