# Supplementary material for: A Phase I First-Time-in-Human, Open-label Study of Nelistotug Administered to Patients with Advanced Solid Tumors
Source: Clin Cancer Res. 2026 Aug 27;32(21):4807–16. doi: 10.1158/1078-0432.CCR-25-4374 (PMC13725324; doi:10.1158/1078-0432.CCR-25-4374)
Supplement: Supplementary file 1 [file ccr-25-4374_supplementary_material1_suppms1.docx]

**Supplementary Appendix**

**Supplementary Methods**

**RNA sequencing**

Baseline pre-treatment and Week 6 post-treatment tumor biopsy samples were obtained for bulk RNA seq analysis (Personalis) from 8 patients with endometrial cancer in Arm B PK/PD. Gene-level RNA-sequencing raw counts and transcripts per million values were provided by Personalis. Differential expression analysis was performed in R using the limma package with the limma-trend method (1). Raw counts were transformed to log2 counts per million with library-size adjustment, and linear models were fitted for each gene. The limma-trend method models the mean-variance relationship through intensity-dependent empirical Bayes moderation, enabling stabilized variance estimates across genes. Genes were ranked by moderated *t*-statistics. Gene set enrichment analysis was then performed using fgseaMultilevel from the R package fgsea (2), which calculates the normalized enrichment score (NES) of any gene signature in a differential gene ranking list. NES values for publicly curated gene signatures were used to generate a heatmap. Signatures with NES >2 were annotated with double asterisks to indicate strong enrichment.

1. Ritchie ME, Phipson B, Wu D, Hu Y, Law CW, Shi W, Smyth GK. Limma powers differential expression analyses for RNA-sequencing and microarray studies. *Nucleic Acids Res* 2015;43(7):e47.

2. Korotkevich G, Sukhov V, Sergushichev A. Fast gene set enrichment analysis. bioRxiv. 2019:doi:10.1101/060012.

**Additional exclusion criteria**

Additional exclusion criteria included relevant toxicity from previous anticancer treatment, including grade ≥3 immune-mediated toxicity that led to treatment discontinuation, additional malignancies that progressed or required active treatment within the last 2 years, uncontrolled or symptomatic central nervous system metastases and/or carcinomatous meningitis, and active autoimmune disease that required systemic disease-modifying or immunosuppressive treatment within the last 2 years.

**Dose-limiting toxicity**

A dose-limiting toxicity (DLT) was defined as an AE that met at least 1 of the criteria listed in the table below and considered by the investigator to be clinically relevant and attributed (definitely, probably, or possibly) to the study intervention during the applicable DLT observation period. An AE considered to be related to the underlying disease under study was not a DLT.

| **Toxicity** | **DLT definition^a^** |
| --- | --- |
| Hematologic | - Grade 4 neutropenia of >7 days in duration or febrile neutropenia - Grade 4 anemia of any duration - Grade 4 thrombocytopenia of any duration or Grade 3 thrombocytopenia with bleeding |
| Nonhematologic | - Any AE (including imAEs) requiring permanent discontinuation of study intervention - ALT ≥3x ULN plus bilirubin ≥2x ULN (>35% direct) or plus INR >1.5, if measured^b^ (possible Hy’s Law) - Grade 2 uveitis, eye pain, or blurred vision that does not resolve with topical therapy within 2 weeks - Grade 2 colitis or diarrhea that persists without resolution to ≤Grade 1 for ≥7 days despite adequate immunosuppressive therapy - ≥Grade 2 immune-mediated endocrine toxicity that requires hormone replacement (except thyroid dysfunction or diabetes that can be medically controlled) - Grade 3 hypersensitivity or infusion-related reaction - ≥Grade 3 nonhematologic clinical toxicity, excluding:   - ≥Grade 3 electrolyte abnormalities that are corrected to ≤Grade 1 or baseline within 72 hours without clinical sequelae   - ≥Grade 3 vital sign abnormalities without clinical significance that are corrected within 48 hours without clinical sequelae   - ≥Grade 3 hyperglycemia that resolves to ≤Grade 1 or baseline within 7 days with adequate therapy   - Grade 3 diarrhea that resolves to ≤Grade 1 or baseline within 7 days with adequate immunosuppressive therapy   - Grade 3 rash that resolves to ≤Grade 2 or baseline within 2 weeks with use of corticosteroids or anti-inflammatory agents per standard of care   - Grade 3 myositis or arthritis that resolves to ≤Grade 1 or baseline within 7 days with adequate immunosuppressive therapy   - Grade 3 tumor flare (defined as local pain, irritation, or rash localized at sites of known or suspected tumor)   - Grade 3 fatigue   - Grade 3 nausea or vomiting resolving to ≤Grade 1 or baseline within 7 days   - Grade 3 peripheral edema resolving to ≤Grade 1 or baseline within 7 days |
| Other | - Any other toxicity considered to be dose-limiting that occurs after the DLT observation period will be considered in decisions regarding dose escalation or the selection of the RP2D - Any other event that in the judgment of the investigator and GSK medical monitor is considered to be a DLT |

^a^Grading according to NCI-CTCAE v5.

^b^Not applicable to participants taking anticoagulants.

AE, adverse event; ALT, alanine transferase; DLT, dose-limiting toxicity; im, immune-modulated; INR, international normalized ratio; NCI-CTCAE, National Cancer Institute-Common Terminology Criteria for Adverse Events; RP2D, recommended phase 2 dose; ULN, upper limit of normal

**Tables**

**Table S1. Primary tumor types in Arms A, B and F**

| **Primary tumor type under study** | **Nelistotug**  **monotherapy**  **(Arm A)**  **N=36** | **Nelistotug + dostarlimab**  **(Arm B)**  **N=33** | **Nelistotug + dostarlimab + belrestotug**  **(Arm F)**  **N=3** | **Total**  **(N=72)** |
| --- | --- | --- | --- | --- |
| Endometrial cancer | — | 14 (42) | — | 14 (19) |
| Head and neck cancer | 9 (25) | 5 (15) |  | 14 (19) |
| Colon cancer | 5 (14) | 4 (12) | 1 (33) | 10 (14) |
| Non-small cell lung cancer | 3 (8) | 1 (3) | 1 (33) | 5 (7) |
| Neoplasm malignant | 3 (8) | 1 (3) | — | 4 (6) |
| Pancreatic carcinoma | 4 (11) | — | — | 4 (6) |
| Salivary gland cancer |  |  |  |  |
| Malignant neoplasm of parotid gland | 4 (11) | — | — | 4 (6) |
| Malignant neoplasm of submandibular gland | 1 (3) | — | — | 1 (1) |
| Breast cancer | 2 (6) | 1 (3) | — | 3 (4) |
| Lip and/or oral cavity cancer |  |  |  |  |
| Squamous cell carcinoma of the oral cavity | 2 (6) | 1 (3) | — | 3 (4) |
| Uterine cancer | 1 (3) | 1 (3) | 1 (33) | 3 (4) |
| Malignant neoplasm of ampulla of vater | 1 (3) | 1 (3) | — | 2 (3) |
| Mesothelioma | 1 (3) | 1 (3) | — | 2 (3) |
| Neuroendocrine carcinoma | 1 (3) | 1 (3) | — | 2 (3) |
| Soft tissue sarcoma | 2 (6) | — | — | 2 (3) |
| Adenocarcinoma of the  gastroesophageal junction | 1 (3) | — | — | 1 (1) |
| Esophageal cancer | — | 1 (3) | — | 1 (1) |
| Malignant melanoma | 1 (3) | — | — | 1 (1) |
| Nasal sinus cancer |  |  |  |  |
| Nasal cavity cancer | — | 1 (3) | — | 1 (1) |
| Oropharynx cancer |  |  |  |  |
| Malignant neoplasm of base of  tongue | — | 1 (3) | — | 1 (1) |
| Ovarian cancer | 1 (3) | — | — | 1 (1) |
| Pharyngeal cancer |  |  |  |  |
| Hypopharyngeal cancer | 1 (3) | — | — | 1 (1) |
| Nasopharyngeal cancer | — | 1 (3) | — | 1 (1) |
| Renal cell carcinoma | 1 (3) | — | — | 1 (1) |
| Renal cancer | — | 1 (3) | — | 1 (1) |
| Tonsil cancer | 1 (3) |  |  | 1 (1) |
| Malignant neoplasm of tonsillar  fossa | — | 1 (3) | — | 1 (1) |
| Vaginal cancer | — | 1 (3) | — | 1 (1) |

**Table S2. Baseline patient and disease characteristics in Arm F**

| **Baseline characteristic** | **Nelistotug + dostarlimab + belrestotug**  **(Arm F)**  **N=3** |
| --- | --- |
| **Age, years^a^** |  |
| Median (range) | 65.0 (50–66) |
| **Sex, n (%)** |  |
| Male | 1 (33) |
| Female | 2 (67) |
| **High-level race, n (%)** |  |
| White | 0 |
| Asian | 3 (100) |
| Black or African American | 0 |
| **Ethnicity, n (%)** |  |
| Hispanic or Latino | 0 |
| Not Hispanic or Latino | 3 (100) |
| **Time since initial diagnosis (years)^b^**  Median (range) | 3 (3–16) |
| **Stage at screening, n/N (%)** |  |
| Ib | 0 |
| IIa | 0 |
| III^c^ | 0 |
| IV^c^ | 3/3 (100) |
| **Primary tumor type, n (%)**^e^ |  |
| Head and neck |  |
| Colon | 1 (33) |
| Pancreatic | – |
| Neoplasms of the parotid gland | – |
| Endometrial | – |
| NSCLC | 1 (33) |
| Uterine | 1 (33) |
| **Number of prior anticancer therapy regimens, n (%)** |  |
| 1 | 0 |
| 2 | 0 |
| 3 | 0 |
| 4 | 1 (33) |
| >4 | 2 (67) |
| Median (range) | >4 (4–>4) |
| **Prior immunotherapy, n (%)** | 0 |

^a^Age is imputed when full date of birth not provided. ^b^Time since initial diagnosis (years) is defined as screening visit year minus initial
diagnosis year. ^c^Includes stages IIIa, IIIc under III, and stages IVa, IVb, IVc under IV.

NSCLC, non-small cell lung cancer.

**Table S3. Representativeness of study patients**

| **Cancer type(s)/ subtype(s)/ stage(s)/ condition** | Advanced solid tumors |
| --- | --- |
| **Considerations related to:** | |
| **Sex** | In 2022, there were approximately 20 million new cancer diagnoses, along with almost 10 million deaths from cancer globally. Of these new diagnoses, 10.3 million were observed in men and 9.7 million in women, with 5.4 and 4.3 million deaths, respectively. Lung cancer and breast cancer have the highest incidence (15.3%, 23.8%) and highest mortality rates (22.7%, 15.4%) in men and women, respectively (1). Sex differences in cancer risk vary significantly with age (2). |
| **Age** | The risk of cancer diagnoses increases significantly with increasing age, with 59% of new diagnoses occurring in the adults ages over 65 years in 2021 (2). |
| **Race/Ethnicity** | In the United States, cancer incidence rates per 100,000 population are highest in the American Indian and Alaskan Native population (497.2), followed by White (476.9), Black (462.0), Hispanic/Latino (362.5), and Asian (303.0) patients (2). |
| **Geography** | Cancer incidence is highest in Asia (49.2%), followed by Europe (22.4%), the Americas (21.1%), Africa (5.9%) and Oceania (1.4%) (1). |
| **Other considerations** | None |
| **Overall representativeness in this study** | In this study, the median age of patients was 63.5 years, with a higher proportion (61%) of female patients. The majority of patients (67%) were White, followed by Asian (26%). This study included 72 patients with a range of advanced solid tumors, therefore the demographics of the current patient population may not be representative of the overall global cancer population. |

(1) Bray F, Laversanne M, Sung H, et al. Global cancer statistics 2022: GLOBOCAN estimates of incidence and mortality worldwide for 36 cancers in 185 countries. *CA Cancer J Clin*. 2024;74:229–263.

(2) Siegel RL, Kratzer TB, Giaquinto AN, et al. Cancer Statistics, 2025. *CA Cancer J Clin*. 2025;75:10-45.

**Table S4. Summary of treatment exposure in Arms A, B, and F**

|  | **Nelistotug**  **Monotherapy Q3W**  **(Arm A)** | | | | | | | | **Nelistotug + dostarlimab 500 mg Q3W**  **(Arm B)** | | | | | **Nelistotug + dostarlimab 500 mg Q3W + belrestotug 400 mg Q3W**  **(Arm F)** |
| --- | --- | --- | --- | --- | --- | --- | --- | --- | --- | --- | --- | --- | --- | --- |
| **Nelistotug dose, mg** | **2.1 (n=3)** | **7 (n=3)** | **21 (n=4)** | **70 (n=3)** | **210 (n=3)** | **700 (n=6)** | **1400 (n=11)** | **1400 PK/PD (n=3)** | **70 (n=3)** | **210 (n=3)** | **700 (n=3)** | **1400**  **(n=11)** | **1400 PK/PD (n=13)** | **1400**  **(N=3)** |
| Nelistotug | | | | | | | | | | | | | | |
| Dose intensity^a^ (mg Q3W), median (range) | 2.1 (1.9–2.1) | 7.0 (6.8–7.0) | 21.1 (21.0–21.5) | 69.4 (68.4–70.0) | 211.3 (209.5–215.1) | 696.1 (683.7–700.0) | 1400.0 (1397.3–1422.6) | 1400.0 (1400.0–1411.2) | 70.0 (70–70) | 203.2 (198–215) | 700.0 (700–700) | 1400 (1291–1400) | 1400 (1140–1434) | 1378.1  (1367–1400) |
| Number of infusions, median (range) | 4.0 (1–12) | 2.0 (1–3) | 2.0 (2–4) | 2.0 (1–11) | 8.0  (2–21) | 4.0  (1–11) | 3.0  (1–25) | 2.0  (2–6) | 2.0 (2–8) | 30.0 (2–34) | 2.0 (2–2) | 3.0  (1–13) | 4.0  (2–16) | 6.0  (2–11) |
| Dostarlimab | | | | | | | | | | | | | | |
| Dose intensity^a^ (mg Q3W), median (range) | - | - | - | - | - | - | - | - | 500.0 (494–500) | 500.0 (463–512) | 500.0 (500–500) | 500.0 (461–500) | 500.0 (407–512) | 492.2  (488–500) |
| Number of infusions, median (range) | - | - | - | - | - | - | - | - | 2.0 (2–6) | 25.0 (2–30) | 2.0 (2–2) | 3.0  (1–13) | 4.0  (2–16) | 6.0  (2–11) |
| Belrestotug | | | | | | | | | | | | | | |
| Dose intensity^a^ (mg Q3W), median (range) | - | - | - | - | - | - | - | - | - | - | - | - | - | 393.8  (391–400) |
| Number of infusions, median (range) | - | - | - | - | - | - | - | - | - | - | - | - | - | 6.0  (2–11) |
| Overall duration of exposure (weeks), median (range) | 12.0 (3.0–40.0) | 6.1 (3.0–9.0) | 6.0 (5.9–11.9) | 6.1 (3.0–33.3) | 23.9 (5.9–63.1) | 12.1 (3.0–33.0) | 8.9  (3.0–75.1) | 6.0  (6.0–17.9) | 6.0 (6.0–24.1) | 93.0 (5.9–108.1) | 6.0 (6.0–6.0) | 9.6 (3.0–42.3) | 11.9 (5.9–49.0) | 18.3  (6.1–33.0) |

^a^Dose intensity is the actual cumulative dose divided by the number of potential 3-week cycles on treatment.

PD, pharmacodynamics; PK, pharmacokinetics; Q3W, every three weeks.

**Table S5. Treatment-emergent adverse events of any grade in Arm F**

| **Preferred term, n (%)** | **Nelistotug + dostarlimab + belrestotug**  **(Arm F)**  **N=3** |
| --- | --- |
| **Any event** | **3 (100)** |
| Constipation | 1 (33) |
| Nausea | 1 (33) |
| Decreased appetite | 1 (33) |
| Pruritus | 1 (33) |
| Rash | 1 (33) |
| Chills | 1 (33) |
| Insomnia | 1 (33) |
| Infusion-related reaction | 1 (33) |
| Lipase increased | 2 (67) |
| Malaise | 2 (67) |

**Table S6. Tumor response (RECIST 1.1) stratified by nelistotug dose in Arms A and B, and Arm F**

|  | **Nelistotug**  **Monotherapy Q3W**  **(Arm A)** | | | | | | | | **Nelistotug + dostarlimab 500 mg Q3W**  **(Arm B)** | | | | | **Nelistotug + dostarlimab 500 mg Q3W + belrestotug 400 mg Q3W**  **(Arm F)** |
| --- | --- | --- | --- | --- | --- | --- | --- | --- | --- | --- | --- | --- | --- | --- |
| **Nelistotug dose, mg** | **2.1 (n=3)** | **7 (n=3)** | **21 (n=4)** | **70 (n=3)** | **210 (n=3)** | **700 (n=6)** | **1400 (n=11)** | **1400 PK/PD (n=3)** | **70 (n=3)** | **210 (n=3)** | **700 (n=3)** | **1400**  **(n=11)** | **1400 PK/PD (n=13)** | **1400**  **(N=3)** |
| **Best overall response, n (%)** | | | | | | | | | | | | | | |
| CR | 0 | 0 | 0 | 0 | 0 | 0 | 0 | 0 | 0 | 1 (33.3) | 0 | 0 | 0 | 0 |
| PR | 0 | 0 | 0 | 0 | 0 | 0 | 0 | 0 | 0 | 1 (33.3) | 0 | 0 | 0 | 0 |
| SD | 2 (66.7) | 1 (33.3) | 1  (25) | 2 (66.7) | 2 (66.7) | 3  (50) | 5 (45.5) | 2  (66.7) | 1 (33.3) | 0 | 0 | 4 (36.4) | 4 (30.8) | 2 (66.7) |
| PD | 1 (33.3) | 2 (66.7) | 3 (75) | 0 | 1 (33.3) | 3 (50) | 5 (45.5) | 1 (33.3_ | 2 (66.7) | 1 (33.3) | 3 (100) | 5 (45.5) | 9 (69.2) | 0 |
| Non-evaluable^a^ | 0 | 0 | 0 | 1 (33.3) | 0 | 0 | 1 (9.1) | 0 | 0 | 0 | 0 | 2 (18.2) | 0 | 1 (33.3) |
| **Confirmed ORR** | | | | | | | | | | | | | | |
| CR+PR, % (95% CI)^b^ | 0 (0, 70.8) | 0 (0, 70.8) | 0 (0, 60.2) | 0 (0, 70.8) | 0 (0, 70.8) | 0 (0, 45.9) | 0 (0, 28.5) | 0 (0. 70.8) | 0 (0, 70.8) | 66.7 (9.4, 99.2) | 0 (0, 70.8) | 0 (0, 28.5) | 0 (0, 24.7) | 0 (0, 70.8) |
| **DCR^c^** | | | | | | | | | | | | | | |
| CR+PR+SD (≥12 weeks), % (95% CI)^b^ | 33.3 (0.8, 90.6) | 0 (0, 70.8) | 0 (0, 60.2) | 33.3 (0.8, 90.6) | 66.7 (9.4, 99.2) | 50 (11.8, 88.2) | 18.2 (2.3, 51.8) | 33.3 (0.8, 90.6) | 33.3 (0.8, 90.6) | 66.7 (9.4, 99.2) | 0 (0, 70.8) | 27.3 (6, 61) | 30.8 (9.1, 61.4) | 33.3 (0.8, 90.6) |

^a^Patients who had at least one post-baseline response assessment and none of the investigator assigned responses qualify to be CR, PR, SD, or PD based on the defined criteria. ^b^Two-sided 95% Exact (Clopper-Pearson) CIs are provided. ^c^DCR is defined as the percentage of patients with a confirmed CR or PR at any time, or SD ≥12 weeks.

CI, confidence interval; CR, complete response; DCR, disease control rate; PD, progressive disease; PR, partial response; Q3W, every three weeks; RECIST 1.1, Response Evaluation Criteria in Solid Tumors, version 1.1; SD, stable disease.

**Table S7. Summary of log-transformed derived serum nelistotug PK parameters: Nelistotug monotherapy (Arm A)**

| **Geometric mean (%CV_b_) PK parameter** | **Visit (Week)** | **Dose (mg)** | | | | | | | |
| --- | --- | --- | --- | --- | --- | --- | --- | --- | --- |
|  |  | **2.1**  **(n=3)** | **7**  **(n=3)** | **21**  **(n=4)** | **70**  **(n=3)** | **210**  **(n=3)** | **700**  **(n=6)** | **1400**  **(n=11)** | **1400 PK/PD**  **(n=3)** |
| AUC_0-τ_, h*µg/mL | 0 | — | — | 1033 (29.0)^c^ | 3112 (37.6^a^ | 14160 (56.1) | 34507 (32.5)^d^ | 80911 (10.1)^f^ | 71129 (8.80)^a^ |
| C_max_, µg/mL | 0 | 0.500 (70.7) | 3.04 (34.0) | 7.17 (31.1) | 19.8 (40.4)^c^ | 89.7 (34.1) | 213 (23.7) | 413 (15.7) | 388 (14.1) |
| C_tau_, µg/mL | 0 | — | — | 0.465 (49.3)^c^ | 2.61 (48.8)^a^ | 11.8 (96.4) | 29.1 (68.0)^d^ | 86.4 (14.5)^f^ | 74.9 (0.755)^a^ |
| RAUC | 3 | — | — | 1.21 (9.84)^c^ | 1.34 (–)^b^ | 1.52 (10.6)^a^ | 1.15 (26.5)^c^ | 1.54 (9.97)^g^ | 1.52 (2.30)^a^ |
|  | 6 | — | — | 1.20 (–)^b^ | 1.80 (–)^b^ | 1.80 (12.3)^a^ | 1.68 (16.5)^a^ | 2.05 (18.0)^c^ | — |
|  | 9 | — | — | — | 1.83 (–)^b^ | 1.95 (–)^b^ | 2.19 (–)^b^ | 2.45 (2.27)^a^ | — |
|  | 12 | — | — | — | 1.90 (–)^b^ | 2.13 (–)^b^ | — | 2.22 (29.2)^c^ | — |
|  | 15 | — | — | — | 1.96 (–)^b^ | 1.77 (17.3)^a^ | 2.11 (–)^b^ | 2.84 (15.5)^a^ | — |
| RC_max_ | 3 | 1.60 (149)^a^ | 0.792 (26.2)^a^ | 0.955 (11.1) | 1.14 (6.24)^a^ | 0.762 (66.6) | 0.905 (48.3)^e^ | 1.22 (18.9)^f^ | 1.26 (19.4) |
|  | 6 | 1.49 (82.3)^a^ | 1.00 (–)^b^ | 0.896 (–)^b^ | 1.35 (–)^b^ | 1.17 (4.95)^a^ | 1.06 (19.9)^d^ | 1.35 (12.7)^h^ | 1.32 (–)^b^ |
|  | 9 | 0.952 (–)^b^ | — | 0.911(–)^b^ | 1.46 (–)^b^ | 1.18 (13.2)^a^ | 1.17 (19.2)^c^ | 1.39 (9.57)^d^ | 1.47 (–)^b^ |
|  | 12 | 5.34 (–)^b^ | — | — | 1.40 (–)^b^ | 1.29 (–)^b^ | 1.10 (15.8)^c^ | 1.37 (19.1)^c^ | 1.57 (–)^b^ |
|  | 15 | 2.75 (–)^b^ | — | — | 1.44 (–)^b^ | 1.14 (14.6)^a^ | 1.36 (–)^b^ | 1.65 (4.10)^a^ | 1.43 (–)^b^ |
| t½, h | 0 | 33.2 (2.10)^a^ | 77.0 (13.8) | 128 (25.5) | 223 (37.4) | 251 (46.4) | 309 (45.2) | 355 (22.8) | 387 (10.1) |
| CL, mL/h | 0 | 60.3 (35.5)^a^ | 25.9 (56.7) | 19.1 (25.8) | 19.3 (42.2) | 11.2 (82.1) | 13.4 (45.8) | 11.5 (18.2) | 12.0 (4.96) |
| V, mL | 0 | 2870 (36.2)^a^ | 2868 (41.8) | 3450 (23.7) | 5541 (24.5) | 3828 (30.6) | 5628 (35.2) | 5594 (14.8) | 6315 (11.2) |
| Median t_max_, h (range) | 0 | 0.57  (0.50–0.58) | 0.50  (0.50–0.53) | 4.63  (0.50–22.93) | 4.52  (0.53–4.58) | 4.55 (0.58–4.57) | 0.67  (0.57–4.67) | 0.58  (0.50–4.90) | 0.58  (0.55–4.67) |

^a^n=2; ^b^n=1; ^c^n=3; ^d^n=4; ^e^n=5; ^f^n=9; ^g^n=7; ^h^n=6.

AUC_0-τ_, area under the curve 0-tau; CL, clearance; C_max_, maximum serum concentration; C_tau_, serum concentration in a dosing interval; CVb, geometric coefficient of variation; PK, pharmacokinetic; RAUC, accumulation ratio based on AUC_0-τ_ ; RC_max_, accumulation ratio based on C_max_; t_1/2_, half-life, t_max_, time to maximum serum concentration; V, volume.

**Table S8. Summary of log-transformed derived serum nelistotug PK parameters: Nelistotug + dostarlimab 500 mg (Arm B)**

|  |  | **Dose (mg)** | | | | | |
| --- | --- | --- | --- | --- | --- | --- | --- |
| **Geometric mean (%CVb) PK parameter** | **Visit (Week)** | **70**  **(n=3)** | **210**  **(n=3)** | **700**  **(n=3)** | **1400**  **(n=11)** | **1400 PK/PD**  **(n=13)** |  |
| AUC_0-τ_, h*µg/mL | 0 | 3205 (59.5) | 14130 (24.8) | 39704 (32.9) | 91774 (34.3)^c^ | 93803 (21.2)^h^ |  |
| C_max_, µg/mL | 0 | 17.6 (42.4) | 73.5 (12.9) | 226 (27.2) | 508 (29.2) | 468 (25.1) |  |
| C_tau_, µg/mL | 0 | 2.65 (57.3) | 14.0 (48.3) | 38.0 (48.8) | 89.7 (42.5)^c^ | 96.6 (48.8)^h^ |  |
| RAUC | 3 | 1.34 (14.6)^a^ | 1.70 (11.8)^a^ | 1.45 (–)^b^ | 1.30 (6.92)^d^ | 1.55 (20.6)^c^ |  |
|  | 6 | 1.93 (–)^b^ | 2.14 (20.1)^a^ | — | 1.60 (18.2)^e^ | 1.74 (15.5)^g^ |  |
|  | 9 | 2.37 (–)^b^ | 2.78 (–)^b^ | — | 1.83 (18.9)^f^ | 1.69 (45.9)^e^ |  |
|  | 12 | 2.50 (–)^b^ | 2.59 (–)^b^ | — | 1.99 (15.1)^g^ | 2.20 (11.5)^a^ |  |
|  | 15 | 2.29 (–)^b^ | 2.37 (26.7)^a^ | — | 2.07 (72.6)^g^ | 2.28 (6.46)^a^ |  |
| RC_max_ | 3 | 1.16 (8.30) | 1.16 (22.5) | 1.11 (6.77) | 1.05 (5.64)^c^ | 1.16 (11.1)^i^ |  |
|  | 6 | 1.17 (–)^b^ | 1.53 (1.96)^a^ | — | 1.07 (10.4)^d^ | 1.15 (23.2)^c^ |  |
|  | 9 | 1.43 (–)^b^ | 1.70 (10.0)^a^ | — | 1.16 (14.2)^e^ | 1.29 (12.1)^d^ |  |
|  | 12 | 1.61 (–)^b^ | 1.53 (–)^b^ | — | 1.10 (15.0)^f^ | 1.32 (12.0)^e^ |  |
|  | 15 | 1.39 (–)^b^ | 1.59 (14.2)^a^ | — | 1.20 (16.4)^f^ | 1.32 (20.2)^f^ |  |
| t½, h | 0 | 259 (19.0) | 295 (30.3) | 285 (26.3) | 304 (34.7) | 355 (38.1) |  |
| CL, mL/h | 0 | 16.4 (58.9) | 10.3 (40.0) | 12.5 (44.8) | 11.0 (40.1) | 9.98 (40.7) |  |
| V, mL | 0 | 5801 (62.9) | 4250 (10.0) | 4960 (20.5) | 4544 (36.6) | 4865 (25.0) |  |
| Median t_max_, h (range) | 0 | 1.05  (0.58–5.22) | 4.58  (4.50– 5.30) | 1.28  (1.25–1.42) | 1.33  (0.60–6.03) | 1.35  (0.55–5.47) |  |

^a^n=2; ^b^n=1; ^c^n=8; ^d^n=6; ^e^n=5; ^f^n=4; ^g^n=3; ^h^n=11; ^i^n=12

AUC_0-τ_, area under the curve 0-tau; CL, clearance; C_max_, maximum serum concentration; C_tau_, serum concentration in a dosing interval; CVb, geometric coefficient of variation; PK, pharmacokinetic; RAUC, accumulation ratio based on AUC_0-τ_; RC_max_, accumulation ratio based on C_max_; t_1/2_, half-life, t_max_, time to maximum serum concentration; V, volume.

**Table S9. Summary of log-transformed derived serum nelistotug PK parameters: Nelistotug + dostarlimab 500 mg + belrestotug 400 mg (Arm F)**

| **Geometric mean (%CVb) PK parameter** | **Visit (Week)** | **n** | **Dose (mg)** |
| --- | --- | --- | --- |
|  |  |  | **1400**  **(n=3)** |
| AUC_0-τ_, h*µg/mL | 0 | 2 | 126, 224 (33.6) |
|  | 12 | 2 | 244, 603 (29.6) |
| C_max_, µg/mL | 0 | 3 | 681 (20.1) |
|  | 12 | 2 | 936 (10.7) |
| C_tau_, µg/mL | 0 | 2 | 122 (96.0) |
|  | 12 | 2 | 336 (30.6) |
| RAUC | 3 | 1 | 1.65 (–) |
|  | 6 | 1 | 1.99 (–) |
|  | 9 | 1 | 1.85 (–) |
|  | 12 | 1 | 1.89 (–) |
| RC_max_ | 3 | 3 | 1.00 (19.0) |
|  | 6 | 2 | 1.35 (3.53) |
|  | 9 | 2 | 1.34 (3.45) |
|  | 12 | 2 | 1.47 (12.4) |
| t½, h | 0 | 3 | 357 (45.7) |
|  | 12 | 2 | 558 (9.10) |
| CL, mL/h | 0 | 3 | 7.56 (54.5) |
|  | 12 | 2 | 2.70 (23.9) |
| V, mL | 0 | 3 | 3704 (16.9) |
|  | 12 | 2 | 2145 (32.4) |
| Median t_max_, h (range) | 0 | 3 | 0.85 (0.80–0.85) |
|  | 12 | 2 | 0.99 (0.82–1.17) |

AUC_0-τ_, area under the curve 0-tau; CL, clearance; C_max_, maximum serum concentration; C_tau_, serum concentration in a dosing interval; CVb, geometric coefficient of variation; PK, pharmacokinetic; RAUC, accumulation ratio based on AUC_0-τ_; RC_max_, accumulation ratio based on C_max;_; t_1/2_, half-life, t_max_, time to maximum serum concentration; V, volume.

**Figure S1. Study design**

^*^PK/PD cohorts restricted to HNSCC, NSCLC, or other tumor types defined based on emerging non-clinical and/or clinical data.

^†^Dose levels for optional back-fill PK/PD cohort(s) were defined based on emerging clinical data.

DLT, dose-limiting toxicity; HNSCC, head and neck squamous cell carcinoma; IV, intravenous; NSCLC, non-small cell lung cancer; PD, pharmacodynamics; PK, pharmacokinetics; Q3W, every 3 weeks; RP2D, recommended phase 2 dose.

**Figure S2. Median serum concentration-time plots of (A) nelistotug as a monotherapy (Arm A) and (B) in combination with dostarlimab 500 mg (Arm B)**

PD, pharmacodynamics; PK, pharmacokinetics.

**Figure S3. Individual patient profile on CD96 RO vs time-course data for nelistotug monotherapy (Arm A) in the dose-escalation cohort and PK/PD cohorts (A) and for the combination with dostarlimab 500 mg (Arm B) in the dose-escalation cohort (B) and in the PK/PD cohort (C)**

**(A)**

**(B)**

**(C)**

**% CD96 Receptor Occupancy**

**1400 mg + 500 mg
 PK/PD**

**CD96RO/CD4+**

**CD96RO/CD56+**

**CD96RO/CD8+**

120

80

40

0

D, Day; HRS, hours; PD, pharmacodynamics; PK, pharmacokinetics; RO, receptor occupancy; W, Week.

**Figure S4. Individual patient data on total soluble CD96 in Arm A**

D, days; HRS, hours; PD, pharmacodynamic; PK, pharmacokinetic; W, Week.

**Figure S5. Tumor pharmacodynamic assessment in Arm B**

In patients treated with nelistotug + dostarlimab (Arm B), RNA seq analysis from paired pre-treatment and Week 6 treatment biopsy samples (n=8) indicated no significant changes from baseline to Week 6 or association with clinical activity (DCR) in CD226 axis relevant genes including *CD96* or *TIGIT* (A). Enrichment of immune activation pathways (B) and activated immune cell signatures (C) was observed in nelistotug + dostarlimab-treated patients at Week 6 compared to baseline in paired biopsy samples.

**In panel B indicate strong normalized enrichments scores (NES).

BL, baseline; DCR, disease control rate (stable disease>12 weeks); W6, Week 6 post-treatment.
